# Splenic Vein Pseudoaneurysm and Rupture Presenting as Maternal Collapse Postpartum: Case Report

**DOI:** 10.1155/crog/7055450

**Published:** 2026-03-04

**Authors:** Reece Burns, Karina Ortman, Jennifer Keomany, Anna Stork-Fury

**Affiliations:** ^1^ Department of Obstetrics and Gynecology, University of Kansas School of Medicine–Wichita, Wichita, Kansas, USA

**Keywords:** case report, intra-abdominal hemorrhage, pregnancy, splenectomy, splenic vein pseudoaneurysm

## Abstract

A multiparous woman at 27 weeks gestation presented with hemodynamic instability and nonreassuring fetal heart tones, prompting emergency cesarean delivery. Intraoperatively, hemoperitoneum was noted without pelvic source. Postoperatively, she deteriorated, necessitating exploratory laparotomy. A ruptured splenic vein pseudoaneurysm was identified and splenectomy was performed. Both mother and infant recovered and were discharged in stable condition. This case report illustrates a rare, life‐threatening cause of intra‐abdominal hemorrhage in pregnancy treated with surgical exploration, emphasizing the need for rapid diagnosis and multidisciplinary management.

**Learning Points**

• Our primary aim is to review and consider nonobstetric causes of intra‐abdominal hemorrhage in pregnant patients with hemodynamic instability when evaluating the abdomen for a source.
• Rapid surgical intervention can be lifesaving.
• Splenic vein aneurysm, although rare, can rupture spontaneously during pregnancy with high maternal–fetal morbidity and mortality reported.
• Multidisciplinary coordination is essential for optimal outcomes in suspected intra‐abdominal hemorrhage.

## 1. Background

Spontaneous intra‐abdominal hemorrhage (SIAH) during pregnancy or postpartum is rare outside of obstetric causes [1, 2]. Common etiologies include uterine rupture, ruptured aneurysms, or visceral organ pathology. Although splenic artery aneurysms are known to rupture during pregnancy with high maternal–fetal mortality, splenic vein rupture is exceedingly rare [1–3]. A recent review identified only three prior cases of splenic vein aneurysm rupture in pregnancy. Given the high risk of mortality, prompt recognition and intervention are critical [1].

## 2. Case Presentation

A 29‐year‐old gravida 3 para 2 woman at 27 + 1 weeks gestation presented to labor and delivery after 8 h of progressively worsening abdominal pain. On radio report from emergency medical services (EMS), she was hypotensive (BP 60 mmHg/palpation on EMS arrival, improved to 90/50 mmHg with fluids) and tachycardic. She denied trauma, vaginal bleeding, or fluid loss. Her history included two prior low transverse cesarean sections and anemia. Her other medical and social history was noncontributory. Body mass index was 35.9 kg/m^2^.

On arrival to labor and delivery triage, vital signs had been stabilized (BP 100/54 mmHg, heart rate [HR] of 89 bpm). On exam, she had a distended abdomen with severe pain focally in her lower abdomen near her prior cesarean scar. Fetal heart tones were difficult to acquire by doppler and a bedside ultrasound demonstrated fetal heart tones in the 80s, prompting emergent transfer to the operating room for emergency cesarean for suspected uterine rupture or abruption.

## 3. Investigations

In the operating room, a repeat bedside ultrasound revealed a live fetus with a normal HR. A thin lower uterine segment was identified without obvious signs of internal hemorrhage. Intravenous access was obtained and labs were drawn which included complete blood counts, comprehensive metabolic panel, fibrinogen, and type and screen. A repeat bedside sonogram was used to assess fetal status which was more reassuring with a HR in the 120 bpm range. Maternal vitals were also stable at this time, so the anesthesia team initiated spinal anesthesia with success. As the patient was repositioned, however, she experienced syncope and was found to be hypotensive. Once positioned on the operating table and secured, blood pressure had improved but bedside sonography demonstrated fetal bradycardia with HR in the 60 bpm range. Anesthesia adequacy was verified and an emergency cesarean was performed.

## 4. Differential Diagnosis

Key considerations in the differential diagnosis for this presentation are as follows:

• Uterine rupture
• Placental abruption
• Intra‐abdominal hemorrhage from visceral source

## 5. Treatment

A viable male infant (APGAR 1/4/5) was delivered. Uterus appeared intact except for a hemostatic uterine window. Five hundred milliliters of clotted blood was noted and cleared from the pelvis. No additional bleeding was identified at this time. Total blood loss was 734 mL. Initial hemoglobin drawn on arrival was 7.7 g/dL. Her comprehensive metabolic panel was unremarkable. She was transferred to labor and delivery for routine recovery.

Two hours postoperatively, she experienced an episode of hypotension (BP 70/41 mmHg) and lightheadedness. Her exam at this time was otherwise unremarkable and 1000 mL of crystalloid and transfusion with 1 unit of packed red blood cell (pRBC) were initiated. Hemoglobin had dropped to 7.6 g/dL on repeat labs. Her vitals improved with volume resuscitation. Once stabilized, her immediate recovery ended, and she was transferred to the postpartum unit with posttransfusion of Hgb 8.2 g/dL.

At 12 h post‐op, the patient′s nurse notified the obstetric team that she was somnolent, and she was evaluated with clinical findings of hypotension, tachycardia, and abdominal distention. She was emergently taken to the operating room for exploratory laparotomy, and during the transition, gynecology–oncology was requested to assist in anticipation of needing cesarean hysterectomy. The blood bank was notified for massive blood transfusion protocol.

Upon exploration, no pelvic or uterine bleeding was found. The upper abdomen was explored and clots in the lesser sac revealed active bleeding. Trauma surgery was emergently consulted intraoperatively, whereas aortic compression was completed to control bleeding. When trauma surgery arrived, they identified a dilated splenic vein at the hilum and performed a splenectomy. Bleeding was controlled but due to the ongoing need for resuscitation and labile vital signs, an EVARREST fibrin patch was placed over the splenic bed, and a temporary abdominal closure (ABThera) was applied after packing the abdomen. Total quantitative blood loss from the procedure was 11,042 mL. She received, in total, 16 units of pRBCs, 20 units of cryoprecipitate, two units of platelets, 250 mL of albumin, and 1 L of lactated ringers.

## 6. Outcome and Follow‐Up

The patient was transferred to ICU and underwent a repeat laparotomy and abdominal closure on postpartum day 2. Her recovery was complicated by acute tubular necrosis, which resolved with supportive cares without dialysis. She was discharged home on postpartum day 13.

Histology: intact spleen with congested hilar vessels and perivascular hemorrhage. No splenic rupture or other pathology identified.

All involved consultants agreed that intraoperative findings and pathology were consistent with a splenic vein pseudoaneurysm.

The neonate was discharged after 53 days with outpatient developmental follow‐up for prematurity and surveillance for possible intraventricular hemorrhage cares. Postoperatively, the patient had uncomplicated outpatient follow‐up and received an appropriate vaccination regimen for the absence of spleen.

## 7. Discussion

This is the third reported case of splenic vein aneurysm or pseudoaneurysm rupture in pregnancy and the fourth overall splenic vein rupture (with or without aneurysm/pseudoaneurysm) that demonstrated maternal and fetal survival [1]. Spontaneous nonobstetric bleeding within the abdominal cavity during pregnancy is exceedingly rare [1, 2]. There are wide ranges of causes of nonobstetric intra‐abdominal bleeding. Although rupture of the splenic artery has been described more frequently, splenic vein rupture is a phenomenon with only 10 prior cases documented, three cases of which led to rupture during pregnancy [1]. Notably, in this case, it is posited that the initial bleeding from the ruptured aneurysm was partially contained by due to compression of the lesser sac by the gravid uterus. Once delivery had been facilitated, slow bleeding was able to continue unchecked to the point of causing symptomatic hemorrhage.

Most intrabdominal aneurysms are found incidentally on imaging and are attributed to arterial rupture [3]. Venous aneurysms, especially in the splenic vein, are rare and often diagnosed only after catastrophic rupture. Etiologies for aneurysm include portal hypertension, vasculitis, or connective tissue disease. Management hinges on early recognition, hemodynamic stabilization, and surgical intervention. If stable or incidentally found on imaging, they can be managed more conservatively with interventional radiologic procedures or expectant management [3]. Comparatively, the more common splenic artery aneurysm presents with nonspecific symptoms, if symptomatic at all [4]. Also infrequent, splenic artery aneurysm and pseudoaneurysm are often found in the background of abdominal trauma or pancreatitis, secondary to pancreatic enzymes in close proximity to digesting the connective tissues surrounding the artery [5]. Splenic vein aneurysm, if found in the stable setting, is often found in the setting of chronic conditions inducing portal hypertension [4]. Both conditions can be difficult to distinguish on workup imaging. Had this patient remained stable to allow for abdominal imaging, a computed‐tomography angiography of the abdomen and pelvis could have helped elicit aneurysm in the region of the splenic hilum and attempt at angiography with intervention could have been utilized for diagnosis and attempt at embolization or endovascular coiling [4, 6]. Definitive management of asymptomatic splenic vein aneurysm is a debated area [7]. Available literature reports a relatively high number of cases ultimately managed with splenectomy; however, serial imaging over multiple years has been demonstrated in some cases [4, 6, 7].

This case highlights the difficulty in diagnosing nonobstetric intra‐abdominal hemorrhage in the presence of fetal bradycardia, often misattributed to placental causes. Multidisciplinary involvement was key to survival, with quick intraoperative consultation to gynecology oncology and trauma surgery providing lifesaving expertise.

## 8. Conclusion

SIAH during pregnancy is a life‐threatening occurrence in pregnancy that can have high morbidity and mortality for both the mother and fetus. This case highlights splenic vein aneurysm and rupture presenting as a rare cause of hemorrhage in the peripartum period and highlights key management concepts such as multidisciplinary team efforts, surgical methods in unstable patients, and rapid recognition of nonobstetric bleeding. Obstetricians should maintain high suspicion nonobstetric intraabdominal bleeding in any emergent cesarean delivery situation for reasons of maternal hemodynamic stability.

## Author Contributions

**Reece Burns:** conceptualization, data curation, writing, original draft, review/editing. **Karina Ortman:** conceptualization and data curation, writing – review and editing. **Jennifer Keomany:** project administration, writing – review and editing. **Anna Stork-Fury:** supervision, data curation, conceptualization, writing – review and editing. All authors were involved in the patient case report and no outside contributions were received.

## Funding

No funding was received for this manuscript.

## Consent

Written informed consent was obtained from the patient for publication of this case report and any accompanying images and consent form is available on request. Our case was fully anonymized.

## Conflicts of Interest

The authors declare no conflicts of interest.

## Data Availability

The data that support the findings of this study are available on request from the corresponding author. The data are not publicly available due to privacy or ethical restrictions.
